# Sign of Pregnancy

**Published:** 1844-03

**Authors:** 


*Sign of Pregnancy.*—The surest token of pregnancy, says Dr. d'Outrepont, is a *dark-bluish hue* of the vaginal mucous membrane, a symptom which is more valuable inasmuch as it is not evanescent or confined to one period, but continues from the period of conception to that of labour. Dr. d'Outrepont has not only met with this appearance uniformly in the human subject, but in different animals which he examined in every period of pregnancy, and which he destroyed to ascertain that no fallacy attended the suspicion of gestation.

*Nene Zeitschr, fur Geburtsh*, xiv., 3.

